# Commentary on structural inequities in global health research funding and practice

**DOI:** 10.3389/fpubh.2026.1827408

**Published:** 2026-08-31

**Authors:** Saralees Nadarajah, Samuel Manda, Queensley C. Chukwudum

**Affiliations:** 1 Department of Mathematics, University of Manchester, Manchester, United Kingdom; 2 Department of Statistics, University of Pretoria, Pretoria, South Africa; 3 Department of Insurance and Risk Management, University of Uyo, Uyo, Akwa Ibom State, Nigeria

**Keywords:** Africa, funding, global south, health, research

## Abstract

The testimonies gathered from researchers across Africa present a damning portrait of persistent structural inequities in how international research partnerships are conceived, funded, and executed. These firsthand accounts, spanning multiple countries and decades of experience, reveal patterns of exploitation, marginalization, and systemic bias that undermine both the integrity of scientific inquiry and the dignity of African researchers. What emerges is not merely a collection of isolated grievances but evidence of deeply embedded colonial-era power dynamics that continue to shape who controls knowledge production about Africa and its peoples. This commentary examines ten representative testimonies from the document, grouped thematically rather than sequentially so that the connections between them emerge clearly, analyzes the systemic issues they illuminate, and proposes concrete recommendations for funders, academic institutions, and researchers committed to equitable partnerships. The voices represented here speak to experiences across the research enterprise, moving from the framing of funding priorities, through the control of financial resources and data, to the marginalization of African intellectual leadership and the distortion of accountability practices.

## 1 Introduction

The testimonies gathered from researchers across Africa present a damning portrait of persistent structural inequities in how international research partnerships are conceived, funded, and executed. These firsthand accounts, spanning multiple countries and decades of experience, reveal patterns of exploitation, marginalization, and systemic bias that undermine both the integrity of scientific inquiry and the dignity of African researchers. What emerges is not merely a collection of isolated grievances but evidence of deeply embedded colonial-era power dynamics that continue to shape who controls knowledge production about Africa and its peoples.

This commentary examines ten representative testimonies from the document, grouped thematically rather than sequentially so that the connections between them emerge clearly, analyzes the systemic issues they illuminate, and proposes concrete recommendations for funders, academic institutions, and researchers committed to equitable partnerships. The voices represented here speak to experiences across the research enterprise, moving from the framing of funding priorities, through the control of financial resources and data, to the marginalization of African intellectual leadership and the distortion of accountability practices.

## 2 Divergence between funding priorities and African realities

The testimonies begin at the level of agenda-setting, where the problems that international funding is willing to support are shown to diverge sharply from the problems African researchers and health systems actually face. This divergence is not incidental but is built into the design of funding mandates themselves, as the two testimonies below illustrate.

### 2.1 Epidemiological mismatch in funding priorities

A leading researcher from Nigeria observes that funding schemes persistently focus on infectious diseases despite epidemiological transitions where non-communicable diseases have overtaken infectious conditions as leading causes of death and disability across Africa. This observation speaks to a fundamental disconnect between global health funding priorities and on-the-ground health realities. The continued emphasis on infectious diseases, while important, reflects donor agendas rather than locally determined health priorities. This misalignment means that African health systems and researchers must continually adapt to external funding streams rather than building sustainable capacity to address their populations' most pressing health needs.

### 2.2 Structural barriers embedded in grant design

This mismatch of priorities is compounded by a deeper structural problem identified by a South African researcher, who offers a critique arguing that funding mandates are fundamentally flawed from inception. Requirements that principal investigators be located outside Africa ensure that money follows control, with African researchers entering collaborations from positions of desperation rather than genuine partnership. This testimony employs powerful language, characterizing current arrangements as modern slavery and exploitation of academically gifted Africans' mindpower. The critique extends beyond individual bad actors to implicate the funding structures themselves as inherently inequitable, setting the stage for the patterns of financial control examined next.

## 3 Financial control and resource capture by northern institutions

Once funding is secured, a second set of testimonies shows that the money itself rarely follows the research it is meant to support. Instead, resources are captured and retained by northern institutions at both the budgeting stage and throughout implementation.

### 3.1 Diversion of grant resources away from performance sites

Another Nigerian researcher, serving as a grant reviewer for major funding bodies, describes being confidentially privy to budgets that allocate substantial personnel and administrative funds to non-African sites that are not the performance locations where data collection occurs. This pattern reveals how funding ostensibly directed toward African health research is significantly diverted to support salaries and infrastructure in high-income countries. The implication is clear: African research institutions serve as data collection sites while the resources that could build their capacity flow elsewhere, perpetuating dependency rather than fostering genuine institutional development.

### 3.2 Financial leakage and inefficiency

This diversion of resources is quantified more precisely by a Zimbabwean researcher, who reports understanding that a significant portion of USAID grant funds remains in the United States before any work begins, with additional depletion through countless journeys by US partners to project countries. The estimate that 25 percent of a million-dollar grant automatically stays with US partners, combined with extensive travel costs, means that only a fraction of intended funding actually reaches the contexts it is meant to benefit. This testimony raises fundamental questions about the efficiency and ethics of funding models that prioritize northern institutional overhead and consultation over direct investment in African capacity.

## 4 Data ownership and extraction

Beyond financial resources, the testimonies reveal that the data generated through African research participation is itself subject to extraction, following the same directional flow away from the continent.

### 4.1 Data colonialism

A South African researcher provides a striking example of how health system data from Malawi is managed through North Carolina, rendering it inaccessible to Malawian researchers. The account describes USAID personnel deployed across African countries, collecting valuable data that is subsequently transferred to Harvard, Seattle, and Washington-based institutions. This practice exemplifies what can only be described as data colonialism, where the raw material of African health information is extracted, processed elsewhere, and converted into academic capital that rarely benefits the communities or researchers who generated it. The researcher notes the irony that even as a Malawian, they cannot access data about their own country's health system.

## 5 Marginalization of African intellectual leadership

A related and recurring theme concerns who is permitted to lead, and to receive credit for, the intellectual work of research. The four testimonies in this section trace this marginalization from the conception of a study, through the bureaucratic systems that formalize it, to the eventual denial of authorship.

### 5.1 Relegation of African principal investigators

A Cameroonian researcher articulates a pernicious pattern where African investigators conceive study designs based on local needs and epidemiological data, yet European institutions holding fiduciary responsibility or established funder relationships are named as principal investigators. The original conceptualizer becomes relegated to co-investigator or field coordinator status, diminishing academic standing and future funding prospects. This testimony explicitly names this practice as intellectual theft and exploitation, where the primary credit accrues to those who did not design the work while the intellectual labor remains invisibilized.

### 5.2 Automated marginalization in application systems

The same Cameroonian researcher describes how this relegation is reinforced by technical infrastructure: applying through a French funding agency portal that automatically designated the French partner as primary contact, routing all communication through them despite the researcher having developed the concept and written the proposal. The award notification and reporting requirements flowed exclusively through the French partner, revealing how even technical infrastructure encodes assumptions about who legitimately leads research. This systemic marginalization operates through seemingly neutral bureaucratic processes that reproduce existing power hierarchies.

### 5.3 Tokenistic inclusion in grant applications

A related but distinct pattern of marginalization is described by a researcher from South Africa, who recounts being approached to provide a curriculum vitae or endorsement so that northern partners could demonstrate African presence in grant applications, only to remain unaware of the funding amount or disposition after awards are made. This practice of tokenistic inclusion reduces African researchers to instrumental roles, their names serving merely to satisfy funder requirements for partnership while excluding them from meaningful participation in financial management or project direction. The testimony connects this practice to the phenomenon of northern researchers using such grants to fund travel to Africa, framed implicitly as combining research with holiday.

### 5.4 Exclusion from authorship after data provision

This marginalization reaches its most stark expression in a testimony from a Nigerian researcher who describes being placed on a grant with northern partners, only to be informed after funding was secured that their services were no longer needed. When data they provided resulted in publications, they received acknowledgment rather than authorship. This practice represents a complete violation of academic norms regarding contribution-based authorship and reveals how collaboration can function as extraction: African researchers provide access and data but are excluded from the intellectual products that confer academic currency.

## 6 Distorted assessment and accountability practices

A final testimony extends these concerns from research partnerships into the adjacent domain of humanitarian assessment, showing that the same dynamics of extraction and exclusion also compromise the evidence base used to guide aid and program decisions.

### 6.1 Distorted assessment practices in Somalia and Somaliland

A Somali researcher provides an extended account of how humanitarian assessments in Somalia and Somaliland are conducted through brief, hotel-based visits by teams temporarily deployed from regional hubs. These assessments rely on pre-selected informants, avoid insecure areas where needs are greatest, and systematically exclude marginalized groups including women, minorities, and persons with disabilities. The testimony describes how institutional incentives discourage critical reporting, as negative findings might delay funding or damage relationships. The result is a cycle where reports validate each other without fresh verification, and serious problems such as aid diversion are reframed as minor implementation challenges.

## 7 Systemic analysis and comparative perspectives

The testimonies presented in this commentary, when considered collectively, reveal interconnected systems of inequity that operate at multiple levels—from agenda-setting to data dissemination. At the level of funding priorities, donor agendas determine what research is possible, often misaligned with local epidemiological realities. At the level of grant implementation, resources flow disproportionately to northern institutions while African partners serve as low-cost data collectors. At the level of knowledge production, data extracted from African contexts is processed and published elsewhere, with African researchers excluded from authorship and intellectual ownership. At the level of accountability, assessment practices systematically produce misleading pictures of program effectiveness while obscuring failures and exclusion.

What makes these patterns particularly insidious is their normalization within institutional structures. Funding portal designs, authorship conventions, overhead allocation formulas, and review processes all encode assumptions that privilege northern institutions and researchers. Individual bad actors exist, but the deeper problem is systemic: the architecture of global health research was built on colonial foundations and has not been fundamentally restructured despite decades of critique.

These dynamics are not unique to Africa. A growing body of literature documents parallel patterns of epistemic injustice, financial extraction, and leadership marginalization in other regions of the Global South. In Latin America, researchers have long critiqued the “scientific dependency” model, where research agendas are shaped by external funders and local knowledge is systematically devalued in favor of northern frameworks (1). More recently, studies of global health partnerships in the region have highlighted how funding conditionalities and bureaucratic requirements effectively exclude local institutions from leadership roles, relegating them to data collection and implementation (2). These accounts echo the Cameroonian and South African testimonies regarding tokenistic inclusion and the relegation of African principal investigators.

Similarly, in South and Southeast Asia, researchers have documented how international collaborations often perpetuate “helicopter research” and “parachute science,” where external partners extract data and publish findings with minimal local authorship or capacity building (3, 4). A systematic review of authorship practices in global mental health research found that papers led by researchers from high-income countries were significantly less likely to include authors from the study setting in senior positions, a pattern that mirrors the Nigerian researcher's experience of exclusion from authorship after data provision (5).

Even in regions with established research infrastructures, such as Eastern Europe and the former Soviet states, scholars have identified enduring asymmetries in research partnerships with Western Europe and North America. These include the diversion of grant resources to northern administrative hubs, a pattern quantified by the Zimbabwean researcher's account of USAID fund leakage, and the privileging of English-language outputs over locally relevant dissemination (6). These comparative examples underscore that the inequities documented in Africa are not aberrations but manifestations of a global political economy of knowledge that systematically advantages institutions and researchers in high-income countries.

However, the African context presents distinct features that amplify these dynamics. The sheer scale of externally funded research relative to domestic investment, the prevalence of vertical disease-specific funding mechanisms, and the historical legacy of colonial governance create a particularly acute dependency structure (7). Unlike some Latin American countries with stronger national research councils or Southeast Asian nations with rapidly growing economies, many African countries lack the fiscal space to mount significant countervailing investments in research capacity (8). This leaves African researchers in a structurally weaker bargaining position, where even well-intentioned partnerships operate within asymmetric power relations that are difficult to renegotiate.

The testimonies also resonate with critiques emerging from within global health and development studies that challenge the “aid effectiveness" paradigm. Recent work has argued that the very structure of donor funding-with its short timeframes, rigid reporting requirements, and emphasis on measurable outputs-is antithetical to building sustainable research capacity and responsive health systems (9). These critiques align with the Somali researcher's account of how institutional incentives discourage critical reporting and perpetuate cycles of superficial assessment. The broader literature on “participation" and “local ownership" in development has repeatedly shown that these concepts are often co-opted to legitimize externally driven agendas rather than genuinely redistribute power (10, 11).

## 8 Recommendations for transformative change

Based on the testimonies, the following recommendations address funders, academic institutions, and researchers committed to equity:

Funding organizations should require that principal investigator status be held by researchers based in the countries where research occurs, with northern partners serving in support roles rather than leadership positions. This structural change would align control and resources with the sites of knowledge production.

Grant budgets should be subject to equity reviews that examine the proportion of funds allocated to African institutions versus northern partners, with transparent justification required when the majority of resources flow outside the research context. Caps on northern institutional overhead and travel expenditures would help ensure funding reaches intended beneficiaries.

Data governance frameworks must be negotiated at the outset of partnerships, establishing that data generated in African contexts remains under the control of African institutions and researchers. Publication policies should require that first and last authorship positions be held by researchers from the countries where data collection occurs, absent compelling justification.

Funding application systems should be redesigned to eliminate automated privileging of northern institutions, with application portals that recognize African institutions as legitimate primary applicants and principal investigators. Language requirements and submission timelines should accommodate diverse contexts rather than assuming northern academic calendars and linguistic competencies.

Assessment and monitoring practices must be reformed to require longer-term presence, independent verification, and systematic engagement with marginalized groups. Confidential mechanisms for community feedback should be established, and reports should be required to document how they have incorporated perspectives from women, minorities, persons with disabilities, and other underrepresented populations.

Institutional incentives should be restructured to reward equitable partnership practices in hiring, promotion, and tenure decisions. Academic institutions in high-income countries should evaluate faculty based on demonstrated commitment to building African research capacity and sharing authorship and funding equitably.

Funder review panels must include substantive representation from African researchers with expertise in local contexts, and reviewer guidance should explicitly address equity considerations. Reviewers should be trained to recognize and penalize tokenistic inclusion practices.

Grant reporting requirements should mandate documentation of how funds were distributed across partner institutions, with public transparency about what proportion reached African organizations and what was spent on northern overhead and travel. This transparency would enable accountability and inform future funding decisions.

Research priority-setting processes should be fundamentally restructured to center African voices and institutions. Rather than donors determining what problems merit attention, funding should support agendas developed through inclusive processes within African research communities and in dialogue with affected populations.

Finally, the research community should establish clear ethical standards for partnership that explicitly name practices such as using African names to secure funding without meaningful inclusion, extracting data without shared authorship, and conducting superficial assessments as violations of research integrity. These standards should carry consequences including ineligibility for future funding.

The testimonies gathered in this document represent decades of accumulated experience and critique from African researchers across the continent. They describe not abstract problems but lived realities of exclusion, exploitation, and marginalization within systems that claim to pursue knowledge and improve health. Responding to these critiques requires more than incremental adjustments; it demands fundamental rethinking of how global health research is conceived, funded, and practiced. The researchers whose voices appear here have done the difficult work of naming these problems. The responsibility now rests with funders, institutions, and northern partners to act on their insights and build genuinely equitable structures for knowledge production about Africa and its peoples.

## Data Availability

The original contributions presented in the study are included in the article/supplementary material, further inquiries can be directed to the corresponding author.
